# The neural correlates of visuo-spatial working memory in children with autism spectrum disorder: effects of cognitive load

**DOI:** 10.1186/1866-1955-6-19

**Published:** 2014-07-15

**Authors:** Vanessa M Vogan, Benjamin R Morgan, Wayne Lee, Tamara L Powell, Mary Lou Smith, Margot J Taylor

**Affiliations:** 1 Diagnostic Imaging and Research Institute, Hospital for Sick Children, 555 University Avenue, Toronto, ON M5G 1X8, Canada; 2 Department of Applied Psychology and Human Development, Ontario Institute for Studies in Education, University of Toronto, Toronto, ON M5S 1V6, Canada; 3 Department of Psychology, University of Toronto, Toronto, ON M5S 1V6, Canada

**Keywords:** Working memory, Autism spectrum disorder, Functional magnetic resonance imaging, Executive function, Cognitive load, Frontal lobe, Parietal lobe

## Abstract

**Background:**

Research on the neural bases of cognitive deficits in autism spectrum disorder (ASD) has shown that working memory (WM) difficulties are associated with abnormalities in the prefrontal cortex. However, cognitive load impacts these findings, and no studies have examined the relation between WM load and neural underpinnings in children with ASD. Thus, the current study determined the effects of cognitive load on WM, using a visuo-spatial WM capacity task in children with and without ASD with functional magnetic resonance imaging (fMRI).

**Methods:**

We used fMRI and a 1-back colour matching task (CMT) task with four levels of difficulty to compare the cortical activation patterns associated with WM in children (7–13 years old) with high functioning autism (*N* = 19) and matched controls (*N* = 17) across cognitive load.

**Results:**

Performance on CMT was comparable between groups, with the exception of one difficulty level. Using linear trend analyses, the control group showed increasing activation as a function of difficulty level in frontal and parietal lobes, particularly between the highest difficulty levels, and decreasing activation as a function of difficulty level in the posterior cingulate and medial frontal gyri. In contrast, children with ASD showed increasing activation only in posterior brain regions and decreasing activation in the posterior cingulate and medial frontal gyri, as a function of difficulty level. Significant differences were found in the precuneus, dorsolateral prefrontal cortex and medial premotor cortex, where control children showed greater positive linear relations between cortical activity and task difficulty level, particularly at the highest difficulty levels, but children with ASD did not show these trends.

**Conclusions:**

Children with ASD showed differences in activation in the frontal and parietal lobes—both critical substrates for visuo-spatial WM. Our data suggest that children with ASD rely mainly on posterior brain regions associated with visual and lower level processing, whereas controls showed activity in frontal lobes related to the classic WM network. Findings will help guide future work by localizing areas of vulnerability to developmental disturbances.

## Background

Approximately 1 in 88 children have an autism spectrum disorder (ASD), and the proportion of children being diagnosed is rising [1]. ASD is a neurodevelopmental disorder classically characterized by social deficits, communicative difficulties and repetitive behaviours [2], with evidence of cognitive and executive function impairment [2-9]. The impaired executive processes may account for many of the profound behavioural manifestations in ASD and contribute to autistic symptomology [6,7]. Emerging literature on cognitive difficulties in ASD, as well as their neural underpinnings, provides evidence for working memory (WM) deficits that are associated with frontal lobe abnormalities, particularly in prefrontal cortical activity [4,10-14].

Previous neuroimaging, electrophysiology and neurochemical studies in ASD have identified atypical white and grey matter volumes [15-18], functional connectivity [10,19], cortical sulcal and gyral anatomy [20], brain lateralization [21], neural perfusion [22] and serotonin synthesis capacity [23] compared to those of typically developing (TD) individuals, with the frontal cortex implicated in a number of these differences [24]. Although there is substantial evidence for developmental anatomical abnormalities of the frontal lobes in ASD, associations with cognitive performance are lacking [25]. It remains to be determined if executive dysfunction in ASD is related to *functional* as well as anatomical frontal lobe aberrations. Further, protracted maturation of the frontal lobes makes the functions they support, such as WM, susceptible to developmental disturbances [26,27] but amenable to therapeutic interventions.

WM allows for the temporary storage, rehearsal and maintenance of information. It is important for learning, social ability [28], academic achievement [29] and many complex cognitive operations [30,31]. Impairments in WM have been reported in ASD, yet our understanding of its development and neural correlates is still very limited in this population. We investigated the neural systems underlying visuo-spatial WM capacity using functional magnetic resonance imaging (fMRI) to determine the role of cognitive load on possible functional differences between children with and without ASD. Cognitive load is a multidimensional construct referring to the processing resources that performing a particular task imposes on one's cognitive system [32]—in the present study, the level of WM task difficulty was used as a measure of cognitive load.

Extant research on the behavioural characterization of WM function in ASD suggests both intact [33-37] WM performance on simple memory tasks, and impaired [33-36,38] performance on more complex tasks, relative to TD individuals. Discrepancies in the literature may be due partly to methodological inconsistencies regarding task choice, comparison groups or population age. Basic WM abilities are intact in high-functioning ASD (see [39] for review). The majority of studies that found significant group differences assessed WM using tasks with increased complexity and/or cognitive load and thereby imposed heavier demands on WM and executive functions [9,34,38]. Further, there is evidence of primarily visuo-spatial WM impairment, whereas verbal WM appears relatively intact in individuals with ASD [35-37].

Only a few studies have examined the neural correlates of WM function in ASD, and no neuroimaging studies of pre-adolescent children exist. Overall, WM processes are largely subserved by the prefrontal and parietal cortices [4,40-44]. Converging literature identifies a broad system of prefrontal, premotor, dorsal cingulate and posterior parietal activation in visual WM tasks see [44], and neuroimaging studies in ASD provide evidence for atypical activity in these regions [10,12,14]. Using an oculomotor visuo-spatial WM task, Luna and colleagues [12] found behavioural impairments in WM, as well as reduced activation in the dorsolateral prefrontal cortex (DLPFC) and posterior cingulate cortex in adults with ASD compared to controls. Interestingly, there was no evidence of impaired activation in other areas known to support WM neural circuitry, such as the anterior cingulate cortex. A study by Koshino et al. [10] demonstrated bilateral activation in the DLPFC in adult controls, whereas adults with ASD showed limited activation in the left, and more right hemisphere recruitment of prefrontal regions, despite the absence of differences in performance accuracy on an n-back letter task. The authors suggested that while typical adults processed letter stimuli using verbal codes, those with ASD employed visual strategies [10], supporting the idea that WM deficits in ASD may be attributed to less efficient processing strategies. In the only study of adolescents, Silk et al. [14] used a visuo-spatial WM mental rotation task, where behavioural performance was similar for ASD and control groups, and observed impaired cortical activation in the frontal lobes in the ASD group, including the anterior cingulate, DLPFC and caudate nucleus, but normal activation in the parietal cortices relative to controls. These findings suggest dysfunctional frontostriatal networks in ASD. More work is needed to understand the neural correlates and developmental trajectory of WM in childhood in ASD, as limited research is available reporting on pre-adolescent children.

One of the most common experimental paradigms used to manipulate cognitive load in studying WM is the ‘n-back’ protocol [10,41,43-47]. In a typical n-back task, participants view a series of stimuli and indicate whether the currently presented stimulus matches one presented ‘n’ (e.g., 0, 1, 2 or 3) trials prior. As difficulty level increases, the number of interfering stimuli between the target and relevant stimulus increases, requiring the utilization of different mental strategies at each level (e.g. 0-back, recognition; 1-back, maintenance; 2-back, maintenance and monitoring). This manipulation consequently increases both memory load *and* executive function demand (i.e. strategy needed to solve the task) in a non-linear fashion from one level to the next, making function-specific changes difficult to quantify and link with brain areas. Therefore, we used a 1-back colour matching task (CMT) [48] which systematically manipulated memory load while keeping executive function constant across all difficulty levels, allowing a direct investigation of the influence of cognitive load on WM. Specifically, executive schemes (i.e. procedural strategies for solving the task) are constant across levels of items in CMT; what varies with each level is the number of relevant items (colours) to be remembered. Difficulty level was parametrically graded based on behavioural age-dependent growth patterns observed using this task in previous work [48]. These observations point to a linear pattern of WM development, which may also be evident in the neural processes across increasing load. Our task is novel, and it captures the brain regions associated with this linear pattern of function across cognitive load in pre-adolescent children. In a previous study from our group, typical adults showed positive linear relations between cortical activity and CMT task difficulty level in areas involved in WM function [49]. Negative linear relations were found in areas typically associated with the default mode network (DMN). The DMN, which has been found in a wide range of neuroimaging studies, is a network of brain regions, including the medial prefrontal cortex, posterior cingulate and inferior parietal lobules, characterized by decreased activation during goal-oriented or attention-demanding tasks [50]. Neurodevelopmental disorders, including ASD, have been associated with abnormal function [51,52] and structure [53] of the DMN that may interfere with cognitive function.

The present study identified and compared the neural activity underlying visuo-spatial WM capacity in children with and without ASD using fMRI, with manipulation of cognitive load. Given the increasing difficulty levels of the current task, we hypothesized that children with ASD would show poorer performance than matched TD controls on behavioural measures of WM. Further, frontal and posterior parietal cortical areas related to visuo-spatial WM capacity would be under-recruited in children with ASD relative to TD controls, and this difference would increase with load. We expected activity to be linearly modulated (positively for WM areas, negatively in default-mode areas) by task difficulty in all children (comparable to previous work with adults), but these trends would be less marked in children with ASD, particularly at higher cognitive loads.

With the protracted maturation of the frontal lobes, and their susceptibility to developmental anomalies, understanding development in these regions is crucial, particularly in populations with frontal lobe abnormalities, as seen in ASD. As previous research has focused on studying WM in adults and adolescents with ASD, there is a critical need to study WM in pre-adolescents with ASD, who exhibit both cognitive and neurological differences. Given the links between social function, school success and executive function ability, investigating the neural bases of WM deficits in children with ASD will contribute to our knowledge of the underlying causes of ASD-related behaviour. Previous research highlights the importance of investigating cognitive impairments in ASD that may arise from brain abnormalities and drive behavioural symptoms [54]. Further, exploring WM correlates will supplement behavioural phenotypes of ASD. This work will help identify the nature of atypical brain development, with future expectations of establishing age-appropriate interventions that can effectively target WM function and, in turn, other symptoms of ASD.

## Methods

### Participants

Seventy-three participants were recruited for this study: 42 children aged 7–13 years with high-functioning ASD, and 31 7–13-year-old TD control children. However, 9 TD children and 23 children with ASD were excluded from the analyses for excessive movement and inadequate task performance or protocol completion. After sex-, IQ- and age-matching, the study sample consisted of 19 children with ASD (3 girls and 16 boys) and 17 controls (4 girls and 13 boys). The groups were matched for age (ASD *M =* 11.05, SD *=* 1.43; TD *M =* 11.12, SD *=* 2.00; *t*_(34)_ = 0.11, ns.), sex (*χ*^2^_(1)_ = 0.34, ns.) and full-scale IQ as determined by the Wechsler Abbreviated Scale of Intelligence-II [55] (ASD *M =* 109.42, SD *=* 15.72; TD *M =* 115.35, SD *=* 9.27; *t*_(34)_ = 1.36, ns.). We substituted the group average IQ for one TD child whose data were missing.

Exclusion criteria for all participants were the presence of any current significant axis I psychiatric comorbidities [2], neurological disorders, medical illnesses, prematurity, uncorrected vision, colour blindness, IQ < 70 as well as standard MRI contraindicators (e.g. ferromagnetic implants). A history of developmental delay, learning disability and attention deficit hyperactivity disorder (ADHD) was used to exclude TD children only; however, these factors were also not current primary diagnoses in the ASD group. Six children with ASD were each on one psychotropic medication (Strattera, Biphentin, Fluoxetine, Concerta, Abilify and Atomoxetine). Their fMRI data were examined in comparison to children with ASD who were not taking medication, and the data did not differ between these subgroups (see Additional file 1). Children were recruited through community support centres, parent support groups, email lists, hospital ads and private schools. Informed consent, clinical and cognitive testing and MRI scanning were performed at the Hospital for Sick Children in Toronto. Experimental procedures were approved by the Research Ethics Board at the Hospital for Sick Children. All children gave informed assent, and the parents provided informed written consent.

Clinical diagnoses of ASD were confirmed in all cases with a combination of expert clinical judgement, clinical records and the Autism Diagnostic Observation Schedule (ADOS) [56], which was administered by a trained individual who maintains inter-rater research reliability. All children completed the backwards digit recall, listening recall, digit recall, mazes memory and block recall subscales of the Working Memory Test Battery for Children (WMTB-C) [57] to supplement behavioural data collected during fMRI tasks. See Table 1 for demographic, neuropsychological and clinical test characteristics.

**Table 1:** Demographic and neuropsychological test characteristics of study sample

**Variables**	**ASD (*N*** **= 19)**		**TD (*N*** **= 17)**		**Significance test**
	**%**	**Mean (SD)**	**%**	**Mean (SD)**	
Demographic data					
Sex (male)	84.21		76.47		*χ*^2^_(1)_ = 0.34, *p* = 0.56
Age		11.05 (1.43)		11.12 (2.00)	*t*_(34)_ = 0.11, *p* = 0.91
IQ		109.42 (15.72)		115.35 (9.27)	*t*_(34)_ = 1.36, *p* = 0.18
ADOS Total		9.68 (2.3)^a^		N/A	
Neuropsychological test data (WMTB-C)					
Digit recall		107.59 (19.53)		116.00 (15.64)	*t*_(34)_ = 1.51, *p* = 0.14
Block recall		95.58 (18.03)		105.35 (20.72)	*t*_(34)_ = 1.51, *p* = 0.14
Mazes memory		95.42 (18.58)		96.35 (23.49)	*t*_(34)_ = 0.13, *p* = 0.90
Listening recall		102.95 (15.77)		116.76 (17.72)	*t*_(34)_ = 2.48, *p* = 0.02*
Backward digit recall		94.05 (18.79)		109.18 (21.09)	*t*_(34)_ = 2.28, *p* = 0.03*

*p < 0.05. ^a^ADOS scores can range from 3–20, with higher scores reflecting greater symptom severity.

### The colour matching task

**Figure 1:**
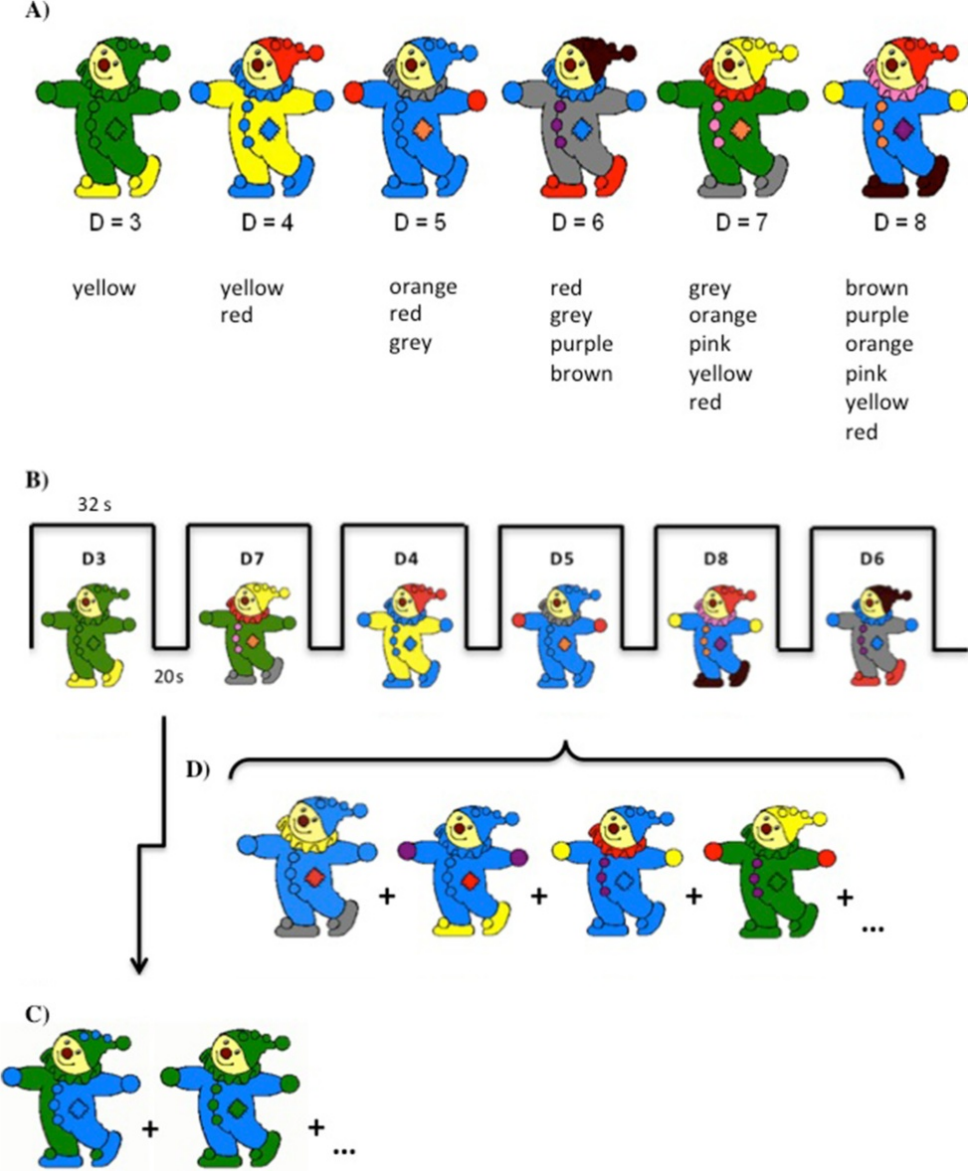
**Protocol description of the colour matching task (CMT). (A)** There were six levels of difficulty where the number of relevant colours (yellow, purple, pink, red, orange, brown and grey) increased by one to increase the difficulty level. Difficulty = (# of colours) + 2. Children were taught to ignore the clown's face, colour location, colour repetition and irrelevant colours (blue and green). **(B)** It was a block design task, where each run consisted of six 32-s task blocks (for each difficulty) followed by 20-s baseline blocks where clowns are presented in only blue and green (ignore). Task blocks were presented pseudo-randomly within each run. **(C)** Example of part of a sequence in a baseline block. Stimuli were presented for 3 s followed by 1-s inter-stimulus cross. Children were instructed to not respond. **(D)** Example of part of a sequence in a task block; children indicated if the current clown was wearing the same or different colours as the previous clown. Stimuli were presented for 3 s followed by a 1-s inter-stimulus fixation cross.

Children were required to attend to coloured figures of a clown presented one at a time in sequence. Children were taught to ignore the clown's face and irrelevant colours (blue and green) and focus on other, relevant colours (yellow, purple, pink, red, orange, brown and grey). Tasks that contain misleading or irrelevant factors that evoke interference have been shown to be a more suitable measure of WM capacity, yielding reliable estimates of developmental growth in adults [48] and children (Powell, Arsalidou, Vogan, and Taylor: Controlled inference and assessments of developmental working memory capacity: Evidence from letter and colour matching tasks, submitted). The number of *‘n’* relevant colours (capacity) in the figures was increased by one for each increase in difficulty level. CMT has two integral functions that require mental attention, in which participants must first actively extract the relevant colours embedded in the clown figure and second check for a possible match with colours of the criterion set. As such, items with *n* (e.g. 1) relevant colours will have difficulty level of *n* + 2 (e.g. 3; Figure 1A). Children indicated after each item whether the relevant colours of the current figure matched those from the immediately preceding figure (i.e. 1-back), disregarding colour location and repetition. Using a keypad with the right hand, children responded in the scanner by pushing a button for ‘same’ when the clown figure was wearing the *same* colours as the previous clown and ‘different’ when the clown figure was wearing *different* colours. All children successfully completed practice trials on a computer outside the scanner with accuracy of 80% or greater.A total of 24 task blocks (168 task trials) and 24 baseline blocks were presented across four runs. Each run consisted of six 32-s blocks, one for each difficulty level (six levels in total); within each block, there was a constant difficulty level, and all difficulty levels were randomized within each run (Figure 1B). The same four runs were presented to all children in the same order. Task blocks consisted of eight stimuli of the same difficulty and alternated with 20-s baseline blocks (Figure 1C), where clowns were coloured only in blue and green, and children were instructed to look at the figures but not respond. Participants had 3 s to view a stimulus and respond, followed by a 1-s inter-stimulus interval where a fixation cross was presented (Figure 1D). The fMRI task took approximately 22 min of scan time.

Performance data were recorded for both accuracy (proportion correct) and reaction time; items were correct if responded correctly within 3 s of stimulus presentation. To ensure adequate task completion, children were excluded if they did not achieve at least 60% accuracy (averaged across four runs) on the easiest two difficulty levels, and also excluded if they did not have at least two out of four runs where 50% or more of the blocks were acceptable in terms of performance (60% accuracy) and motion. Motion was deemed acceptable if children moved less than 1.5 mm from their median head position in at least 60% of the volumes within a task block. See the fMRI preprocessing section below for a description of displacement calculations. A 60% accuracy criterion was chosen because while we could be sure that children were performing better than chance (50%), it was not too stringent. Motion parameters were also entered into the fMRI preprocessing pipeline.

### Image acquisition

All imaging data were acquired using a 3 T Siemens Trio MRI scanner with a 12-channel head coil. Head stabilization and motion restriction were achieved with foam padding. The structural scan was a high-resolution T1-weighted 3D MP-RAGE image (Sagittal; FOV = 192 × 240 × 256 mm; 1 mm isometric voxels; TR/TE/TI/FA = 2,300/2.96/900/9), which was used as an individual anatomical reference for the functional images. During structural image acquisition, children watched a movie of their choice using MR-compatible goggles and earphones. Functional images were acquired with single-shot echo planar imaging sequence (Axial; FOV = 192 × 192; Res = 64 × 64; 30 slices 5 mm thick; 3 × 3 × 5 mm voxels; TR/TE/FA = 2,000/30/70). Visual stimuli for the functional task (CMT) were displayed on MR-compatible goggles. Children responded to trials using a dual button MR-compatible keypad. Stimuli were displayed and performance was recorded using the software *Presentation* (Neurobehavioral Systems Inc., Berkeley, CA, USA).

### Behavioural data analyses

#### CMT

Accuracy on difficulty levels 7 and 8 (D7 and D8) was poor for both TD children (D7, *M =* 0.57, SD *=* 0.03; D8, *M =* 0.53, SD = 0.02) and children with ASD (D7, *M =* 0.53, SD *=* 0.02; D8, *M* = 0.54, SD = 0.03); thus analyses of only the first four difficulty levels (D3 to D6) were completed. Accuracy and response times were calculated for each difficulty level by averaging across runs for each group. Data were analyzed using repeated measures factorial ANOVAs, with group (ASD and TD) as a between subject factor and difficulty level (D3, D4, D5 and D6) as a within subject factor.

#### WMTB-C

Standardized scores on the subscales were compared across group using *t* tests to determine if there were differences between ASD and TD children on these neuropsychological measures of WM.

### fMRI data analyses

Image preprocessing of functional data was performed using a combination of standard AFNI [58] and FMRIB's Software Library (FSL) [59] tools. The first three volumes of each run were discarded for scanner stabilization. After slice timing and motion correction, data were smoothed using a 6-mm FWHM Gaussian kernel, temporally filtered (lower and upper cutoff frequencies of 0.01 and 0.2 Hz, respectively) and converted to percent signal change from the baseline volumes. Before group-level analyses, images were registered to the Montreal Neurological Institute (MNI) 152 template. The maximum Euclidean displacement (MD) travelled by any voxel within the brain was calculated from the six rigid body transformation parameters for each volume. This MD metric was used to flag volumes with unacceptable motion, as described above. The average MD for each subject was used to explore group differences in head motion. Although more motion was found in children with ASD (*M* = 0.58 mm, SD = 0.54 mm) than TD children (*M* = 0.29 mm, SD = 0.22 mm), *t*_(34)_ = 2.07, *p* = 0.05, both groups had minimal average motion of under 0.60 mm. To control for motion, MD was also included as a covariate of no interest in the GLM.

Data were analyzed with the FSL fMRI Expert Analysis Tool (FEAT) [60]. Data were fit first to a block-design general linear model convolved with a gamma function to model haemodynamic response, using the task parameters (D3 to D6). To examine areas that linearly modulated as a function of difficulty, linear trend analyses were conducted from D3 to D6 using fixed-effects higher level modelling. Individual results were then averaged across runs for each subject in a second-level analysis. Between-group comparisons were carried out using FMRIB's Local Analysis of Mixed Effects-1 (FLAME 1) [59] to obtain an accurate between-subject variance estimation, which increased our ability to detect real activation [60]. Significant activations were reported using cluster-based thresholding determined by Z > |2.3| and a corrected cluster significance threshold of *p* < 0.05. Regions of interest (ROIs) were selected from the local maxima of areas showing significant group differences between TD and ASD groups in the linear trend analyses. Average percent signal change and standard error scores were extracted from spherical ROIs (6 mm radius) centred about the local maxima of group difference maps, and the mean peak cluster signal change for each group was plotted as a function of difficulty to further examine activation patterns across cognitive load.

## Results

### Behavioural data

**Figure 2:**
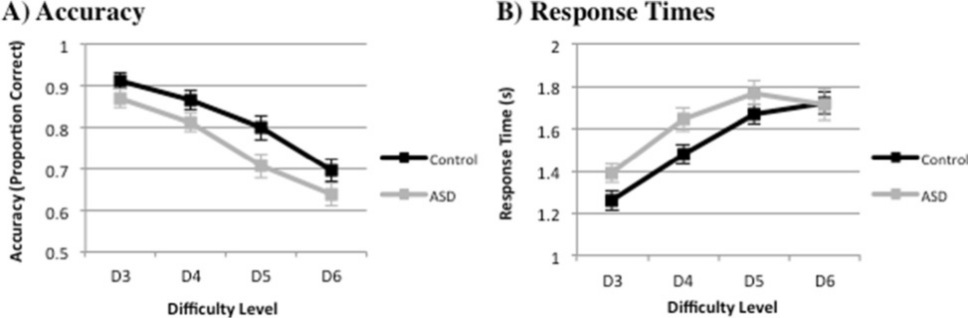
**CMT behavioural performance. (A)** Mean proportion correct for D3 to D6 and standard error bars. TD children were significantly more accurate than children with ASD at D5 only. **(B)** Mean response times for D3 to D6 and standard error bars. There were no significant differences between TD and ASD groups in response times across difficulty levels.

There was a significant effect of group on accuracy (Figure 2A), *F*_(1, 34)_ = 5.15, *p* = 0.03, *η*^2^ = 0.13, which was driven by TD children (*M =* 0.80, SD *=* 0.12) performing more accurately than ASD children (*M =* .71, SD *=* 0.12) only at D5 (*t*_(34)_ = 2.30, *p* = 0.03). As such, comparisons of brain activity between control and ASD groups were made under comparable accuracy scores across most levels. There was a significant main effect of difficulty on accuracy, *F*_(3, 34)_ = 53.87, *p <* 0.001, *η*^2^ = 0.61, with performance accuracy decreasing as a function of difficulty level in both groups. *Post hoc* pairwise comparisons, adjusted for multiple comparisons using Bonferroni, revealed that accuracy on the most difficult level was significantly different from each other at *p* < 0.05, the exceptions being between D5 and D6 in the ASD group (Table 2), and between D3 and D4, and between D4 and D5 in TD children (Table 2). We also ran a supplementary analysis, inputting performance as a covariate. There were no areas of activation that correlated with performance in either group, suggesting that findings were not confounded by performance per se. Also, there were no significant differences in performance across runs in the TD (*F*_(3, 48)_ =1.21, *p* = 0.32) or ASD (*F*_(3, 45)_ = 0.50, *p* = 0.69) group, and therefore patterns of performance did not reflect fatigue across time.

**Table 2:** Differences in CMT accuracy (proportion correct) across difficulty

	**Differences in CMT accuracy**					
	**D3**		**D4**		**D5**	
	**MD**	**SE**	**MD**	**SE**	**MD**	**SE**
Children with ASD						
D4	0.06**	0.02				
D5	0.17***	0.03	0.11**	0.03		
D6	0.23***	0.03	0.17***	0.03	0.07	0.03
TD Children						
D4	0.05	0.02				
D5	0.11*	0.03	0.07	0.02		
D6	0.22***	0.03	0.17***	0.03	0.11**	0.03

*p < 0.05, **p < 0.01, ***p < 0.001, corrected for multiple comparisons using Bonferroni. *Post hoc* ANOVA tests using accuracy scores for (A) children with ASD and (B) TD children. *D3 to D6* difficulty levels 3 to 6, *MD* mean difference, *SE* standard error.

Overall, response times increased with increasing difficulty in TD children but only increased up until D5 in children with ASD (Figure 2B). There was no main effect of group on response times (*F*_(1, 34)_ = 2.33, *p* = 0.14), but there was a main effect of difficulty level, *F*_(3, 34)_ = 54.60, *p* < 0.001, *η*^2^ = 0.62. *Post hoc* comparisons in the ASD group showed that response times differed between difficulty levels, except between D4 and D6, and between D5 and D6 (Table 3). In TD children, response times significantly differed between difficulty levels, except between D4 and D6 and D5 and D6 (Table 3).

**Table 3:** Differences in CMT response times (seconds) across difficulty levels

	**Differences in CMT response times**					
	**D3**		**D4**		**D5**	
	**MD**	**SE**	**MD**	**SE**	**MD**	**SE**
Children with ASD						
D4	−0.25***	0.05				
D5	−0.38***	0.05	−0.13**	0.03		
D6	−0.33**	0.07	−0.08	0.07	0.05	0.05
TD Children						
D4	−0.22***	0.03				
D5	−0.41***	0.04	−0.19***	0.03		
D6	−0.47***	0.07	−0.25**	0.06	−0.06	0.05

*p < 0.05, ** *p* < 0.01, ***p < 0.001, corrected for multiple comparisons using Bonferonni. *Post hoc* ANOVA tests using response times for (A) children with ASD and (B) TD children. *D3 to D6* difficulty levels 3 to 6, *MD* mean difference, *SE*, standard error.

For the WMTB-C, TD children had significantly higher scores on the listening recall (*t*_(34)_ = 2.48, *p* < 0.05) and backward digit recall (*t*_(34)_ = 2.28, *p* < 0.05) subtests than children with ASD. The groups did not differ on any other subtests (see Table 1 for scores on the WMTB-C).

### fMRI Data

#### Task-related activation within groups

**Figure 3:**
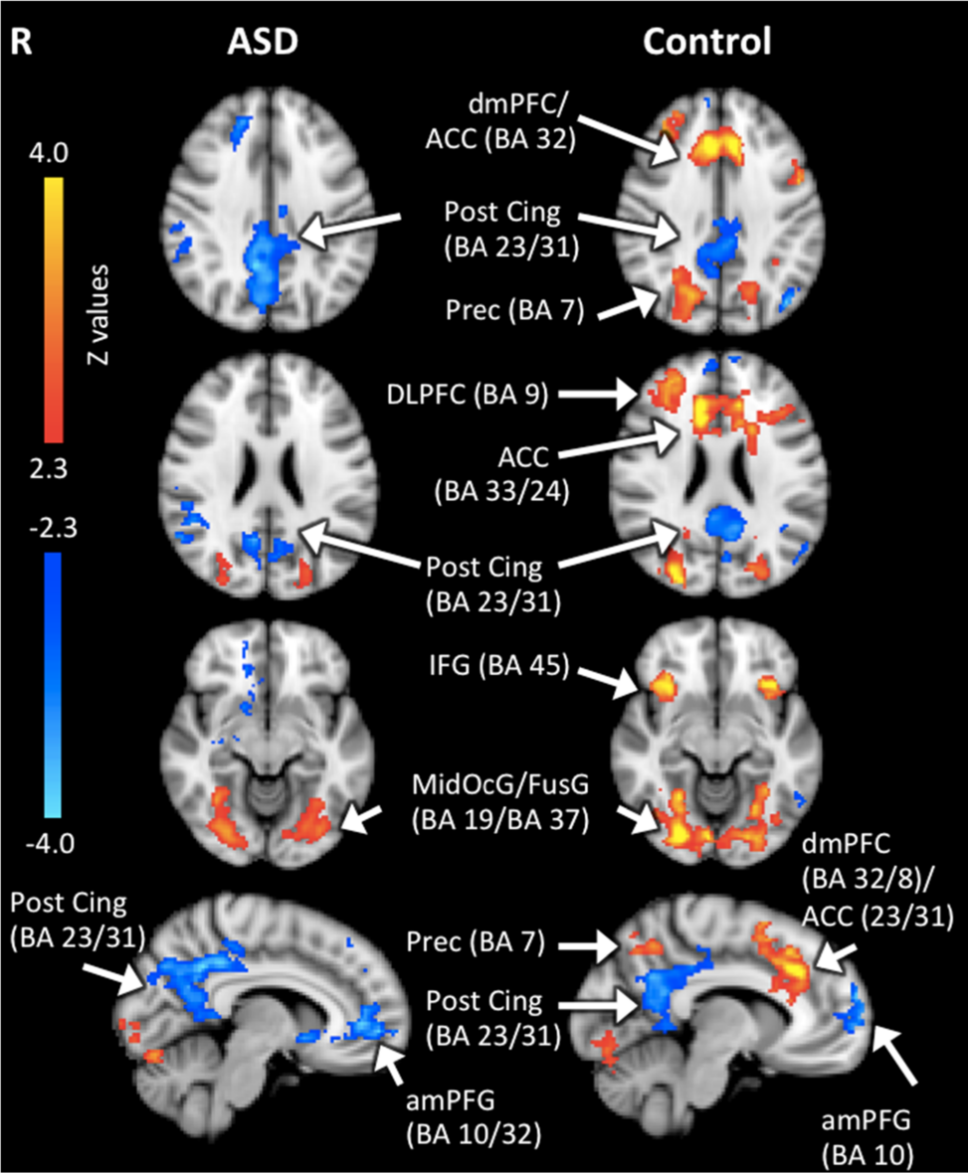
**Group activation maps for the linear trend analyses in ASD and TD groups during CMT.** Significant activations using cluster-based thresholding determined by Z > |2.3| and a corrected cluster significance threshold of *p* = 0.05. Areas in red depict regions of increasing activation as a function of difficulty (i.e. positive linear relations between cortical activity and task difficulty level), and areas in blue depict regions of decreasing activation (i.e. negative linear relations between cortical activity and task difficulty level). *dmPFC* dorsal medial prefrontal cortex, *Post Cing* posterior cingulate cortex, *Prec* precuneus, *DLPFC* dorsolateral prefrontal cortex, *ACC* anterior cingulate cortex, *IFG* inferior frontal gyrus, *MidOcG* middle occipital gyrus, *FusG* fusiform gyrus, *amPFG* anterior medial prefrontal gyrus.

The primary objectives of the fMRI analyses were to investigate the pattern of brain activity exhibited as a function of cognitive load (i.e. difficulty level) and determine if this pattern differed in children with and without ASD. Linear trend analyses (D3 to D6) showed that while some brain areas increased in activity as a function of difficulty level, others decreased. ‘Increasing activation’ refers to an *increase* in BOLD signal with increasing load (i.e. positive linear relations between cortical activity and task difficulty level) and ‘decreasing activation’ refers to a *decrease* in BOLD signal with increasing load (i.e. negative linear relations between cortical activity and task difficulty level). As shown in Figure 3, magnitude of the signal change increased with difficulty in TD children, particularly between D5 and D6, in bilateral fusiform (BA37), precuneus (BA7), inferior frontal gyri (BA45), right DLPFC (BA9) and bilateral dorsal cingulate/dorsal medial prefrontal cortex (B32/8) extending to the anterior cingulate (B33/24). In the bilateral middle occipital gyrus (BA19), a positive linear change in activation with increasing task difficulty was seen up to D6 (Table 4). Activation found in the bilateral posterior cingulate (BA 23/31) and anterior medial prefrontal gyrus (BA10) decreased (i.e. showed a negative linear relation) as a function of difficulty level (Table 4).

**Table 4:** Linear trend analyses across difficulty levels: TD group

**Linear trend analyses across difficulty levels for TD children**								
	**Voxels**	**MNI Coordinates**			***Z *** **value**	***P *** **value**	**Hem.**	**Region**
		***x***	***y***	***z***				
Regions where activation increases with difficulty (increasing BOLD signal)	8885	−26	−90	16	5.04	1.88 × 10^−21^	L	Middle occipital gyrus
	X	24	−82	−10	4.93		R	Fusiform/lingual gyrus
	X	24	−62	50	4.86		R	Precuneus
	X	38	−84	10	4.78		R	Middle occipital gyrus
	X	−16	−64	56	4.25		L	Precuneus
	X	−20	−82	−14	4.22		L	Fusiform/lingual gyrus
	6845	32	26	0	4.97	7.26 × 10^−18^	R	Inferior frontal gyrus
	X	−32	24	−4	4.95		L	Inferior frontal gyrus
	X	8	28	32	4.94		R	Anterior cingulate cortex
	X	32	42	22	4.04		R	Dorsolateral prefrontal cortex
	X	−12	30	24	3.90		L	Anterior cingulate cortex
Regions where activation decreases with difficulty (decreasing BOLD signal)	1808	−6	−56	30	−3.68	9.54 × 10^−7^	L	Posterior cingulate gyrus
	X	4	−40	34	−3.47		R	Posterior cingulate cortex
	725	−42	−72	36	−3.69	3.29 × 10^−3^	L	Middle temporal gyrus
	629	8	48	42	−3.76	7.91 × 10^−3^	R	Medial frontal gyrus
	X	−6	62	20	−3.61		L	Medial frontal gyrus

Results from linear trend analyses from D3 to D6 for TD children. Areas that increased as a function of difficulty level (A) are associated with WM and visuo-spatial processing, whereas areas that decreased as a function of difficulty level (B) are associated with the default mode network. MNI coordinates represent the peak *Z* value of the cluster; *X* peak local maximas within cluster.

Children with ASD did not show the same trends in activation, particularly in the frontal brain regions. A positive linear relation between cortical activity and task difficulty was seen only in the posterior brain regions, including the bilateral middle occipital (BA19) and fusiform gyri (BA37, Table 5). Similar to TD children, activation in the posterior cingulate (BA23/31) and medial prefrontal gyrus (BA10/32) decreased (i.e., showed a negative linear relation) as a function of difficulty level in children with ASD (Table 5).

**Table 5:** Linear trend analyses across difficulty levels: ASD group

**Linear trend analyses across difficulty levels for children with ASD**								
	**Voxels**	**MNI Coordinates**			***Z *** **value**	***P *** **value**	**Hem.**	**Region**
		***x***	***y***	***z***				
Regions where activation increases with difficulty (increasing BOLD signal)	1816	28	−52	−14	3.61	8.94 × 10^−7^	R	Fusiform gyrus
	X	26	−84	12	3.42		R	Middle occipital gyrus
	1449	−26	−88	8	3.52	1.09 × 10^−5^	L	Middle occipital gyrus
	X	−20	−68	−14	3.17		L	Fusiform gyrus
Regions where activation decreases with difficulty (decreasing BOLD signal)	2210	10	−50	36	−3.80	5.96 × 10^−8^	R	Posterior cingulate cortex
	X	−6	−68	24	−2.91		L	Posterior cingulate cortex
	1308	12	54	0	−4.06	3.02 × 10^−5^	R	Medial frontal gyrus
	X	−10	40	8	−2.98		R	Medial frontal gyrus/Anterior cingulate cortex
	642	18	36	44	−3.64	7.00 × 10^−3^	R	Superior frontal gyrus
	509	60	−56	−2	−3.63	2.89 × 10^−3^	R	Middle temporal gyrus

Results from linear trend analyses from D3 to D6 for children with ASD. Areas that increased as a function of difficulty level (A) are associated with WM and visuo-spatial processing, whereas areas that decreased as a function of difficulty level (B) are associated with the default mode network. MNI coordinates represent the peak Z value of the cluster; *X* peak local maximas within cluster.

#### Between-group differences in task-related activation

**Figure 4:**
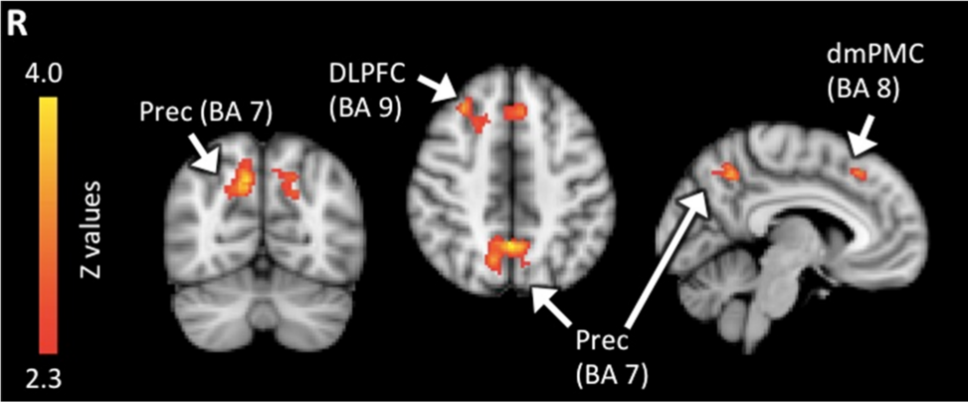
**Results from between-group comparisons.** Significant activations using cluster-based thresholding determined by *Z* > 2.3 and a corrected cluster significance threshold of *p* = 0.05. Areas in red depict regions where the control children showed greater positive linear relations between cortical activity and task difficulty level than the ASD group. There were no areas where the ASD group showed greater linear activation trends across difficulty level in the negative or positive direction than controls. *Prec* precuneus, *DLPFC* dorsolateral prefrontal cortex, *dmPMC* dorsal medial premotor cortex.

**Figure 5:**
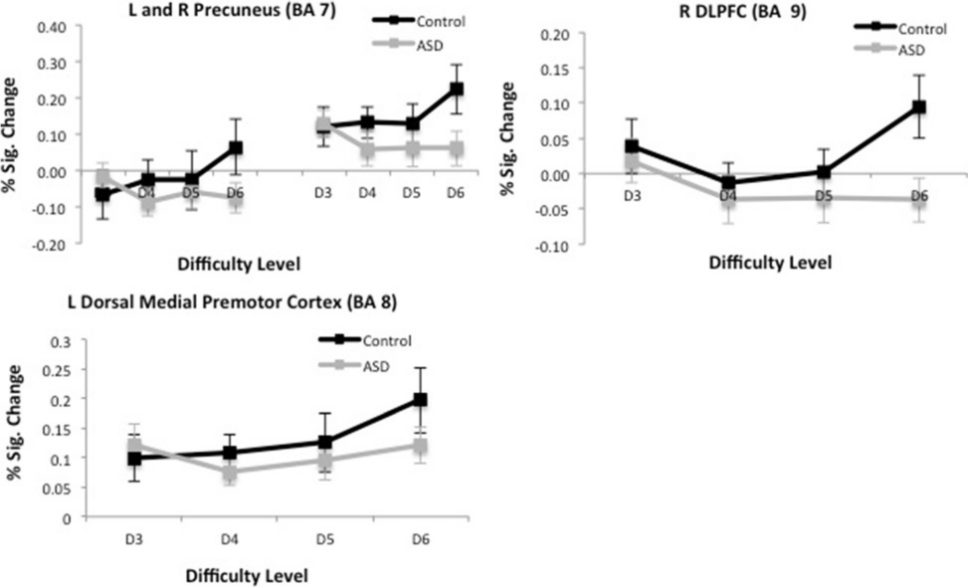
**Mean peak cluster percent signal changes and standard error.** As a function of difficulty between task difficulty and baseline conditions in areas where children with ASD significantly differed from TD children in the linear trend analyses. *DLPFC* dorsolateral prefrontal cortex.

Between-group analyses of each difficulty level separately showed no significant differences (see Additional file 2). However, there were significant group effects in *linear activation trends* as a function of difficulty level. As shown in Figure 4, three regions demonstrated group differences in activation. These areas included the bilateral precuneus (BA7), right DLPFC (BA9) and left dorsal medial premotor cortex (BA8) (Table 6). In these regions, TD children showed significant positive linear relations between cortical activity and difficulty level, particularly between D5 and D6, whereas children with ASD did not show these trends between D5 and D6. The magnitude of the signal change in frontal and parietal areas increased largely with the most difficult level when TD children tried to meet WM demand, but children with ASD failed to show this increase. See Figure 5 for graphs of percent signal change with standard error for ROIs of brain regions that showed significantly different linear patterns between TD and ASD groups.

**Table 6:** Regions of significant differences between TD and ASD groups

**Voxels**	**MNI Coordinates**			***Z *** **value**	***P *** **value**	**Hem.**	**Region**
	***x***	***y***	***z***				
810	0	−56	46	3.93	1.56 × 10^−3^	L	Precuneus
X	10	−66	40	3.81		R	Precuneus
796	34	34	40	3.65	1.76 × 10^−3^	R	Dorsolateral prefrontal cortex
X	−6	26	46	3.13		L	Dorsal medial premotor cortex

Results from between group comparisons of the linear trend analyses from D3 to D6. All regions reported are areas in which TD children showed greater positive linear relations between cortical activity and difficulty level (i.e. increasing BOLD signal with increasing difficulty) than children with ASD. There were no areas where children with ASD showed greater linear relations between cortical activity and difficulty level than TD children. MNI coordinates represent the peak *Z* value of the cluster; *X* peak local maximas within cluster.

## Discussion

This is the first study to examine the neural correlates of visuo-spatial WM in pre-adolescent children with ASD relative to TD children. Whereas children with and without ASD did not differ in brain activation during WM function irrespective of load, between-group differences were observed in the linear activation trends across difficulty level. In other words, children with and without ASD differ in how they *modulate* WM processes during tasks that increase in difficulty level. Using a task that isolated cognitive load on WM, we observed positive linear relations between cortical activity and task difficulty level in prefrontal and parietal regions, particularly between the highest difficulty levels, in TD children that were significantly different from activation trends seen in children with ASD. These areas included bilateral precuneus, right DLPFC and left medial premotor cortices. Other areas known to underlie WM function, including the anterior cingulate and inferior frontal gyri, followed this same pattern but the linear activation trends did not differ significantly between TD and ASD subjects, likely due to subject variability. Overall, TD children demonstrated an opposing system of cognitive processes where areas related to task difficulty (frontal regions) increased in activity and areas associated with the brain's DMN (posterior cingulate and anterior medial frontal gyrus) decreased in activity with increasing cognitive demand; this pattern of activation was absent in children with ASD. There were significant differences in performance on only two verbal WM subtests of the WMTB-C. Further, TD and ASD groups performed similarly on the CMT (except for a small difference at D5), suggesting that the findings are not confounded by behavioural differences.

TD children showed increased recruitment of both frontal and posterior parietal regions as a function of cognitive load, whereas the ASD group showed increased recruitment of only the posterior visual regions, including the left and right fusiform and middle occipital gyri. Our observed pattern of findings in children with ASD are consistent with findings in adults with ASD who also relied on posterior regions related to low-level cognition during WM tasks, rather than areas of high level-cognition, such as in the prefrontal cortices [10]. More activation in occipital-temporal areas in ASD has been proposed to reflect visually based processing styles and a tendency to rely primarily on visual features and details of objects, rather than on WM [61]. These findings are in line with the enhanced perceptual functioning model [62] that suggests individuals with ASD display superior activation of visuo-perceptual regions in association with a reduced activation in higher order frontal areas. The present study extends this literature in adults by highlighting that this processing style appears in young children with ASD as well. The same pattern of activation has also been found in socially relevant tasks [63,64]. Although greater posterior activation may be adequate for visuo-spatial processing, it is likely inefficient for more complex cognitive operations, such as language comprehension [19] and WM [10], which would have implications for the social and executive deficits typically observed in ASD. Less extensive use of prefrontal areas may be the result of early abnormal prefrontal development [15,16] and connectivity [10,19] reported in individuals with ASD.

The finding of stable activation with increasing cognitive load within the prefrontal cortex may be most significant for ASD due to the crucial role of frontal regions in WM and executive function [10,43,44,65-67]. Similar to adults [10,12] and adolescents [14] with ASD, we found that pre-adolescent children with ASD demonstrated different activation patterns in the DLPFC (BA 9) during WM function. Specifically, in the DLPFC, TD children showed a graded increase in activity from D3 to D6, whereas activity within this region did not increase at the same magnitude in children with ASD. The DLPFC is believed to play a critical role in holding information ‘online’ [26] and mediating strategic organization and data compression processes [68,69], hence its sensitivity to increasing cognitive demand in ours and other studies [68-71]. Stable activation across difficulty level in this area suggests that young children with ASD may fail to use appropriate organizational strategies, such as ‘chunking’ methods, which facilitate WM by simplifying cognitive load/demand. Furthermore, normative studies have demonstrated that with increasing age, individuals rely more on DLPFC in WM processes [43,66,72], suggesting that specialization of this region for WM coincides with structural maturation across development. It is possible that atypical abnormal growth patterns in ASD in the frontal cortex, the DLPFC in particular [15], adversely impacts its functional integrity; these speculations require further research.

Failure to increase recruitment of the precuneus across difficulty level in ASD subjects is also of interest, given evidence supporting parietal involvement in addition to the prefrontal cortex during spatial WM processing [44,66,70,73]. The precuneus forms part of the proposed occipito-parietal network, or alternatively the visual ‘dorsal pathway’, responsible for spatial visual processing (e.g. object location) [74]. Conversely, the occipito-temporal, or visual ‘ventral pathway’, includes the fusiform gyri and is critical to object identification (e.g. colour and shape) [74]. Given that CMT was a visuo-spatial task with gradual increases in WM that needed to be processed, areas responsive to both spatial search (occipito-parietal regions) and categorization (occipito-temporal regions) should show increased recruitment across cognitive load. Although group differences in fusiform activity were absent, we observed a significant difference in the precuneus; TD children showed positive linear relations between cortical activity and difficulty level, particularly between D5 and D6, whereas the ASD group did not show this linear change. Impaired parietal activity in pre-adolescent children with ASD lends support to the proposed dorsal stream deficits within this population as well as in other developmental disorders [75-78], while ventral processing is relatively intact. Furthermore, normative developmental studies suggest that the dorsal pathway has a more protracted maturational trajectory than the ventral stream [66], and WM fMRI studies demonstrate greater parietal [4,73] but less fusiform recruitment [66] with age. Thus, spared fusiform/occipital activity and abnormal parietal functioning in our ASD group may reflect immature WM processing typically seen in very young TD children. However, it is not clear whether the recruitment of mature neural substrates underlying WM processes is delayed, persistently weak or arrested. Future longitudinal work will help clarify the developmental path of WM neural circuitry in ASD.

## Conclusions

Overall, the current study fills a significant gap in our knowledge of neural substrates implicated in visuo-spatial WM functioning among pre-adolescent children and how they differ in children with ASD. Similar to adults, we found that children with ASD show prefrontal and parietal system abnormalities and tend to rely on posterior brain regions associated with lower level cognitive processing. The present study also converges on the growing body of literature proposing dorsal stream deficits in visuo-spatial processing in this population. This study contributes new information on WM differences in school-aged children with and without ASD, demonstrating that even in pre-adolescents, there are significant differences in brain activation patterns during WM processing with increasing cognitive load that differentiate the groups. In light of similar task performance between groups, it is also important to entertain the idea that higher order processing may not be mandatory in ASD with tasks that can be processed efficiently using a perceptual processing approach because of their proposed enhanced perceptual functioning [62]. Conversely, higher order control may be necessary in TD children who do not have such perceptual expertise. Although data may support this argument, ASD is generally considered a network/brain disorder, and what is interesting is how networks in children with ASD differ from TD children with complex tasks. Group effects were driven by the most difficult level analyzed, which may be due to strategies used by TD children necessary to encode the increased number of colours in the highest task load (i.e. meet high WM demand) that children with ASD are unable to employ. Similarly, previous research reports behavioural differences with tasks that have more complex WM demands e.g. [9,34,39].

It is important to consider the limitations of the current study when interpreting results. With comparable CMT behavioural performance between ASD and TD groups, we eliminated performance as a confounding factor. Consequently, our sample was less representative of low-functioning individuals with ASD, and thus, results are generalizable to higher functioning individuals only. Future fMRI research is required to understand WM function across various levels of functioning and a range of symptoms. In addition, given our choice of control subjects (TD children), findings can only provide information about differences from the norm. Comparisons to other atypical populations who share similar cognitive but different clinical profiles (e.g. ADHD) [5] will further our understanding about the neural patterns that are *unique* to ASD, potentially explaining characteristic behaviour in this complex group. Lastly, results are also limited by the relatively small sample size, due to assessing a complex cognitive ability while scanning, which greatly increased the amount of children's data that was not usable.

The findings from the current study have a number of significant implications despite these limitations. Several researchers have stressed the importance of executive cognitive skills for social function [5,79], a core deficit of ASD. However, there remains a gap in knowledge regarding the link between neuropsychopathology and clinical symptoms of autism. With respect to our findings of atypical neural activity underlying WM processes in ASD, this may impair the ability to hold information ‘online’ that may affect one's ability to evaluate and select appropriate responses during peer interactions, translating into socially inappropriate behaviour. Social behaviour requires complex cognitive processing, further highlighting the importance of our results of increasing group differences in brain activity with increases in complexity. Future neuroimaging studies could work towards understanding the relation between ASD symptomology and neural activation patterns associated with WM processing. Overall, our findings will help guide future longitudinal work by localizing areas of vulnerability to developmental disturbances and allow health care providers to carefully monitor their development. Developmental information will allow us to identify the nature and timing of atypical development, which is critical in establishing age-appropriate cognitive or pharmacological remediation for WM function and behavioural deficits in ASD.

## Competing interests

The authors declare that they have no competing interests.

## Authors’ contributions

VV is responsible for recruitment, data acquisition, analysis and interpretation as well as drafting the manuscript. BM participated in fMRI analysis and revising manuscript. WL developed the fMRI analysis pipelines and helped with experimental design and statistical analyses. MS advised on patient testing, study design and revising manuscript. TP assisted with recruitment, data acquisition and revising manuscript. MT initiated the study and participated in design, analyses and revising manuscript. All authors read and approved the final manuscript.

## Supplementary Material

Additional file 1 **fMRI data for medicated and non-medicated children with ASD.** Percent signal change as a function of difficulty between task difficulty and baseline conditions in children with ASD who were on medication versus those who were not. Areas of the brain shown are from regions where children with ASD significantly differed from TD children in the linear trend analyses. Potential differences between medicated and non-medicated children with ASD were also examined statistically using the FSL FEAT, and no significant differences were found between children with ASD who were and were not on medication. However, due to low *N* (only six subjects on medication), this statistical test may not be reliable. Therefore scatter plots were created to visually examine the data for significant group differences, and this reaffirmed that medication does not appear to affect the findings. Click here for file

Additional file 2 **Individual group activation maps for children with ASD and typically developing children at all levels (D3 to D6) of CMT.** Significant activations using cluster-based thresholding determined by *Z* > |2.3| and a corrected cluster significance threshold of *p* = 0.05. Areas in red and blue depict regions with significantly higher and lower BOLD signal than baseline, respectively. Between-group comparisons showed no areas of significant difference between children with and without ASD at any single difficulty level. Click here for file

## References

[B1] Centers for Disease Control and Prevention (CDC) Prevalence of autism spectrum disorders–autism and developmental disabilities monitoring network, United States, 2008 MMWR Morbidity and Mortal Weekly Report 2012 61 3 1 44 22456193

[B2] American Psychiatric Association Diagnostic and statistical manual of mental disorders 2013 5 Arlington, VA: American Psychiatric Publishing

[B3] Barnard L Muldoon K Hasan R O’Brien G Stewart M Profiling executive dysfunction in adults with autism and comorbid learning disability Autism 2008 12 125 141 18308763 10.1177/1362361307088486

[B4] Greene CM Braet W Johnson KA Bellgrove MA Imaging the genetics of executive function Biol Psychol 2008 79 30 42 18178303 10.1016/j.biopsycho.2007.11.009

[B5] Happe F Booth R Charlton R Hughes C Executive function deficits in autism spectrum disorders and attention-deficit/hyperactivity disorder: examining profiles across domains and ages Brain Cognition 2006 61 25 39 16682102 10.1016/j.bandc.2006.03.004

[B6] Hill EL Executive dysfunction in autism Trends Cogni Sci 2004 8 26 32 10.1016/j.tics.2003.11.003 14697400

[B7] Joseph RM Neuropsychological frameworks for understanding autism Int Rev Psychiatr 1999 11 309 324 10.1080/09540269974195 PMC1351137 16467917

[B8] Luna B Doll SK Hegedus SJ Minshew NJ Sweeney JA Maturation of executive function in autism Biol Soc 2007 61 474 481 10.1016/j.biopsych.2006.02.030 16650833

[B9] Russo N Flanagan T Iarocci G Berringer D Zelazo PD Burack JA Deconstructing executive deficits among persons with autism: implications for cognitive neuroscience Brain Cognition 2007 65 77 86 17825970 10.1016/j.bandc.2006.04.007

[B10] Koshino H Carpenter PA Minshew NJ Cherkassky VL Keller TA Just MA Functional connectivity in an fMRI working memory task in high-functioning autism Neuroimage 2005 24 810 821 15652316 10.1016/j.neuroimage.2004.09.028

[B11] Koshino H Kana RK Keller TA Cherkassky VL Minshew NJ Just MA fMRI investigation of working memory for faces in autism: visual coding and underconnectivity with frontal areas Cereb Cortex 2007 18 289 300 17517680 10.1093/cercor/bhm054 PMC4500154

[B12] Luna B Minshew NJ Garver KE Lazar NA Thulborn KR Eddy WF Sweeny JA Neocortical system abnormalities in autism An fMRI study of spatial working memory Neurology 2002 59 834 840 12297562 10.1212/wnl.59.6.834

[B13] O’Hearn K Asato M Ordaz S Luna B Neurodevelopment and executive function in autism Dev Psychopathol 2008 20 1103 1132 18838033 10.1017/S0954579408000527

[B14] Silk TJ Rinehart N Bradshaw JL Tonge B Egan G O’Boyle MW Cunnington R Visuospatial processing and the function of prefrontal-parietal networks in autism spectrum disorders: a functional MRI study Am J Psychiat 2006 163 1440 1443 16877661 10.1176/ajp.2006.163.8.1440

[B15] Carper RA Courchesne E Localized enlargement of the frontal cortex in early autism Biol Psychiatry 2005 57 126 133 15652870 10.1016/j.biopsych.2004.11.005

[B16] Hazlett HC Poe MD Gerig G Smith RG Piven J Cortical gray and white brain tissue volume in adolescents and adults with autism Biol Psychiatry 2006 59 1 6 16139816 10.1016/j.biopsych.2005.06.015

[B17] Herbert MR Ziegler DA Deutsch CK O’Brien LM Lange N Bakardjiev A Hodgson J Adrien KT Steele S Makris N Kennedy D Harris GJ Caviness VS Dissociations of cerebral cortex, subcortical and cerebral white matter volumes in autistic boys Brain 2003 126 1182 1192 12690057 10.1093/brain/awg110

[B18] Mak-Fan KM Taylor MJ Roberts W Lerch JP Measures of cortical grey matter structure and development in children with autism spectrum disorder J Autism Dev Disord 2012 42 419 427 21556969 10.1007/s10803-011-1261-6

[B19] Just MA Cherkassky VL Keller TA Minshew NJ Cortical activation and synchronization during sentence comprehension in high-functioning autism: evidence of underconnectivity Brain 2004 127 1811 1821 15215213 10.1093/brain/awh199

[B20] Levitt JG Blanton RE Smalley S Thompson PM Guthrie D McCracken JT Sadoun T Heinichen L Toga AW Cortical sulcal maps in autism Cereb Cortex 2003 13 728 735 12816888 10.1093/cercor/13.7.728

[B21] McPartland J Dawson G Webb SJ Panagiotides H Carver LJ Event-related brain potentials reveal anomalies in temporal processing of faces in autism spectrum disorder J Child Psychol Psyc 2004 45 1235 1245 10.1111/j.1469-7610.2004.00318.x 15335344

[B22] Zilbovicius M Garreau B Samson Y Remy P Barthelemy C Syrota A Lelord G Delayed maturation of the frontal cortex in childhood autism Am J Psychiat 1995 152 248 252 7840359 10.1176/ajp.152.2.248

[B23] Chandana SR Behen ME Juhász C Muzik O Rothermel RD Mangner TJ Chakraborty PK Chungani HT Chungani DC Significance of abnormalities in developmental trajectory and asymmetry of cortical serotonin synthesis in autism Int J Dev Neurosci 2005 23 171 182 15749243 10.1016/j.ijdevneu.2004.08.002

[B24] Lainhart JE Advances in autism neuroimaging research for the clinician and geneticist Am J Med Genet 2006 142C 33 39 16419098 10.1002/ajmg.c.30080

[B25] Griebling J Minshew NJ Bodner K Libove R Bansal R Konasale P Keshavan MS Hardan A Dorsolateral prefrontal cortex magnetic resonance imaging measurements and cognitive performance in autism J Child Neuro 2010 25 856 863 10.1177/0883073809351313 PMC3428128 20097663

[B26] Powell KB Voeller KK Prefrontal executive function syndromes in children J Child Neuro 2004 19 785 797 10.1177/08830738040190100801 15559894

[B27] Sowell ER Thompson PM Leonard CM Welcome SE Kan E Toga AW Longitudinal mapping of cortical thickness and brain growth in normal children J Neurosci 2004 24 8223 8231 15385605 10.1523/JNEUROSCI.1798-04.2004 PMC6729679

[B28] Dennis M Agostino A Roncadin C Levin H Theory of mind depends on domain-general executive functions of working memory and cognitive inhibition in children with traumatic brain injury J Clin Exp Neuropsyc 2009 31 835 847 10.1080/13803390802572419 19221924

[B29] Alloway TP Working memory, but not IQ, predicts subsequent learning in children with learning difficulties Eur J Psychol Assess 2009 25 92 98

[B30] Baddeley A Working memory Science 1992 255 556 559 1736359 10.1126/science.1736359

[B31] Engle RW Tuholski SW Laughlin JE Conway AR Working memory, short-term memory, and general fluid intelligence: a latent-variable approach J Exp Psychol Gen 1999 128 309 10513398 10.1037//0096-3445.128.3.309

[B32] Paas FG Van Merriënboer JJ Variability of worked examples and transfer of geometrical problem-solving skills: a cognitive-load approach J Educ Psychol 1994 86 122

[B33] Benetto L Pennington BF Rogers SJ Intact and impaired memory functions in autism Child Dev 1996 67 1816 1835 8890510

[B34] Minshew NJ Goldstein G The pattern of intact and impaired memory functions in autism J Child Psychol Psyc 2001 42 1095 1101 10.1111/1469-7610.00808 11806691

[B35] Russell J Jarrold C Henry L Working memory in children with autism and with moderate learning difficulties J Child Psychol Psyc 1996 37 673 686 10.1111/j.1469-7610.1996.tb01459.x 8894948

[B36] Ozonoff S Strayer DL Further evidence of intact working memory in autism J Autism Dev Disord 2001 31 257 263 11518480 10.1023/a:1010794902139

[B37] Steele SD Minshew NJ Luna B Sweeney JA Spatial working memory deficits in autism J Autism Dev Disord 2007 37 605 612 16909311 10.1007/s10803-006-0202-2

[B38] Williams DL Goldstein G Carpenter PA Minshew NJ Verbal and spatial working memory in autism J Autism Dev Disord 2005 35 747 756 16267641 10.1007/s10803-005-0021-x

[B39] Williams DL Minshew NJ Goldstein G Boucher J, Bowler D Memory within a complex information processing model of autism Memory in autism 2008 New York: Cambridge University Press

[B40] Baddeley A Working memory: looking back and looking forward Nat Rev Neurosci 2003 4 829 839 14523382 10.1038/nrn1201

[B41] Carlson S Martinkappi S Rama P Salli E Korvenoja A Aronen HJ Distribution of cortical activation during visuospatial n-back tasks as revealed by functional magnetic resonance imaging Cereb Cortex 1998 8 743 752 9863701 10.1093/cercor/8.8.743

[B42] Fuster JM Prefrontal neurons in networks of executive memory Brain Res Bull 2000 52 331 336 10922510 10.1016/s0361-9230(99)00258-0

[B43] Kwon H Reiss AL Menon V Neural basis of protracted developmental changes in visuo-spatial working memory Proc Natl Acad Sci U S A 2002 99 13336 13341 12244209 10.1073/pnas.162486399 PMC130634

[B44] Owen A McMillan KM Laird AR Bullmore E N-back working memory paradigm: a meta-analysis of normative functional neuroimaging studies Hum Brain Map 2005 25 46 59 10.1002/hbm.20131 PMC6871745 15846822

[B45] Kirchener WK Age differences in short-term retention of rapidly changing information J Exp Psychol 1958 55 352 358 13539317 10.1037/h0043688

[B46] Cohen JD Perlstein WM Braver TS Nystrom LE Noll DC Jonides J Smith EE Temporal dynamics of brain activation during a working memory task Nature 1997 386 604 608 9121583 10.1038/386604a0

[B47] Nelson CA Monk CS Lin J Carver LJ Thomas KM Truwit CL Functional neuroanatomy of spatial working memory in children Dev Psychol 2000 36 109 116 10645748 10.1037//0012-1649.36.1.109

[B48] Arsalidou M Pascual-Leone J Johnson J Misleading cues improve developmental assessment of working memory capacity: the color matching tasks Cognitive Dev 2010 25 262 277

[B49] Arsalidou M Pascual-Leone J Johnson J Morris D Taylor MJ A balancing act of the brain: activations and deactivations driven by cognitive load Brain Behav 2013 3 273 285 23785659 10.1002/brb3.128 PMC3683287

[B50] Whitfield-Gabrieli S Ford JM Default mode network activity and connectivity in psychopathology Annu Rev Clin Psychol 2012 8 49 76 22224834 10.1146/annurev-clinpsy-032511-143049

[B51] Assaf M Jagannathan K Calhoun VD Miller L Stevens MC Sahl R O`Boyle JG Schultz RT Pearlson GD Abnormal functional connectivity of default mode sub-networks in autism spectrum disorder patients Neuroimage 2010 53 247 256 20621638 10.1016/j.neuroimage.2010.05.067 PMC3058935

[B52] Kennedy DP Redcay E Courchesne E Failing to deactivate: resting functional abnormalities in autism Proc Natl Acad Sci U S A 2006 103 8275 8280 16702548 10.1073/pnas.0600674103 PMC1472462

[B53] Uddin LQ Menon V Young CB Ryali S Chen T Khouzam A Minshew NJ Hardan AY Multivariate searchlight classification of structural magnetic resonance imaging in children and adolescents with autism Biol Psychiatry 2011 70 833 841 21890111 10.1016/j.biopsych.2011.07.014 PMC3191298

[B54] Frith U Mind blindness and the brain in autism Neuron 2001 32 969 979 11754830 10.1016/s0896-6273(01)00552-9

[B55] Wechsler D Wechsler intelligence scale for children 2003 San Antonia, TX: Psychological Corporation

[B56] Lord C Risi S Lambrecht L Cook EH Leventhal BL DiLavore PC Pickles A Rutter M The autism diagnostic observation schedule-generic: a standard measure of social and communication deficits associated with the spectrum of autism J Autism Dev Disord 2000 30 205 223 11055457

[B57] Pickering S Gathercole S Working memory test battery for children 2001 London, UK: Pearson Assessment

[B58] Cox RW AFNI: Software for analysis and visualization of functional magnetic resonance neuroimages Comput Biomed Res 1996 29 162 173 8812068 10.1006/cbmr.1996.0014

[B59] Worsley KJ Jezzard P, Matthews PM, Smith SM Statistical analysis of activation images Ch 14, in functional MRI: an introduction to methods 2001 Oxford, UK: Oxford University Press

[B60] Woolrich MW Jbabdi S Patenaude B Chappell M Makni S Behrens T Beckmann C Jenkinson M Smith S Bayesian analysis of neuroimaging data in FSL Neuroimage 2009 45 S173 S186 19059349 10.1016/j.neuroimage.2008.10.055

[B61] Ring HA Baron-Cohen S Wheelwright S Williams SCR Brammer M Andrew C Bullmore ET Cerebral correlates of preserved cognitive skills in autism Brain 1999 122 1305 1315 10388796 10.1093/brain/122.7.1305

[B62] Mottron L Dawson M Soulières I Hubert B Burack J Enhanced perceptual functioning in autism: an update, and eight principled of autistic perception J Autism Dev Disord 2006 36 27 43 16453071 10.1007/s10803-005-0040-7

[B63] Hubl D Bolte S Feineis-Matthews S Lanfermann H Federspiel A Strik W Poustka F Dierks T Functional imbalance of visual pathways indicates alternative face processing strategies in autism Neurology 2003 61 1232 1237 14610126 10.1212/01.wnl.0000091862.22033.1a

[B64] Pierce K Müller RA Ambrose JB Allen G Courchesne E Face processing occurs outside the fusiform ‘face area’ in autism: evidence from functional MRI Brain 2001 124 2059 2073 11571222 10.1093/brain/124.10.2059

[B65] Duncan J Owen AM Common regions of the human frontal lobe recruited by diverse cognitive demands Trends Neurosci 2000 23 475 483 11006464 10.1016/s0166-2236(00)01633-7

[B66] Scherf KS Sweeney JA Beatriz L Brain basis of developmental change in visuospatial working memory J Cognitive Neurosci 2006 18 1045 1058 10.1162/jocn.2006.18.7.1045 16839280

[B67] Wager TD Smith EE Neuroimaging studies of working memory Cognitive Affective Behav Neurosci 2003 3 255 274 10.3758/cabn.3.4.255 15040547

[B68] Rypma B Berger JS D’Esposito M Performance on prefrontal cortical activity J Cognitive Neurosci 2002 14 721 731 10.1162/08989290260138627 12167257

[B69] Bor D Duncan J Wiseman RJ Owen AM Encoding strategies dissociate prefrontal activity from working memory demand Neuron 2003 37 361 367 12546829 10.1016/s0896-6273(02)01171-6

[B70] Rypma B Prabhakaran V Desmond JE Glover GH Gabrieli JD Load-dependent roles of frontal brain regions in the maintenance of working memory Neuroimage 1999 9 216 226 9927550 10.1006/nimg.1998.0404

[B71] Finn AS Sheridan MA Kam CLH Hinshaw S D’Esposito M Longitudinal evidence for functional specialization of the neural circuit supporting working memory in the human brain J Neurosci 2010 30 11062 11067 20720113 10.1523/JNEUROSCI.6266-09.2010 PMC2952435

[B72] Klingberg T Forssberg H Westerberg H Increased brain activity in frontal and parietal cortex underlies the development of visuospatial working memory capacity during childhood J Cognitive Neurosci 2002 14 1 10 10.1162/089892902317205276 11798382

[B73] Olesen PJ Nagy Z Westerberg H Klingberg T Combined analysis of DTI and fMRI data reveals a joint maturation of white and grey matter in a fronto-parietal network Cognitive Brain Res 2003 18 48 57 10.1016/j.cogbrainres.2003.09.003 14659496

[B74] Ungerleider LG Mishkin M Ingle DJ I, Goodale MA, Mansfield RJW Two cortical visual systems Analysis of visual behavior 1982 Cambridge, Mass: The MIT Press 549 586

[B75] Pellicano E Gibson LY Investigating the functional integrity of the dorsal visual pathway in autism and dyslexia Neuropsychologia 2008 46 2593 2596 18501932 10.1016/j.neuropsychologia.2008.04.008

[B76] Pellicano E Gibson L Maybery M Durkin K Badcock DR Abnormal global processing along the dorsal visual pathway in autism: a possible mechanism for weak visuospatial coherence? Neuropsychologia 2005 43 1044 1053 15769490 10.1016/j.neuropsychologia.2004.10.003

[B77] Spencer J O’Brien J Riggs K Braddick O Atkinson J Wattam-Bell J Motion processing in autism: evidence for a dorsal stream deficiency Cognitive Neurosci Neuropsychol 2000 11 2765 2766 10.1097/00001756-200008210-00031 10976959

[B78] Grinter EJ Maybery MT Badcock DR Vision in developmental disorders: is there a dorsal stream deficit? Brain Res Bull 2010 8 147 160 20211706 10.1016/j.brainresbull.2010.02.016

[B79] Beauchamp MH Anderson V SOCIAL: an integrative framework for the development of social skills Psychol Bull 2010 136 39 64 20063925 10.1037/a0017768

