# Supplementary figures and images for: The neural correlates of visuo-spatial working memory in children with autism spectrum disorder: effects of cognitive load
Source: J Neurodev Disord. 2014 Jul 15;6(1):19. doi: 10.1186/1866-1955-6-19 (PMC4107490; doi:10.1186/1866-1955-6-19)

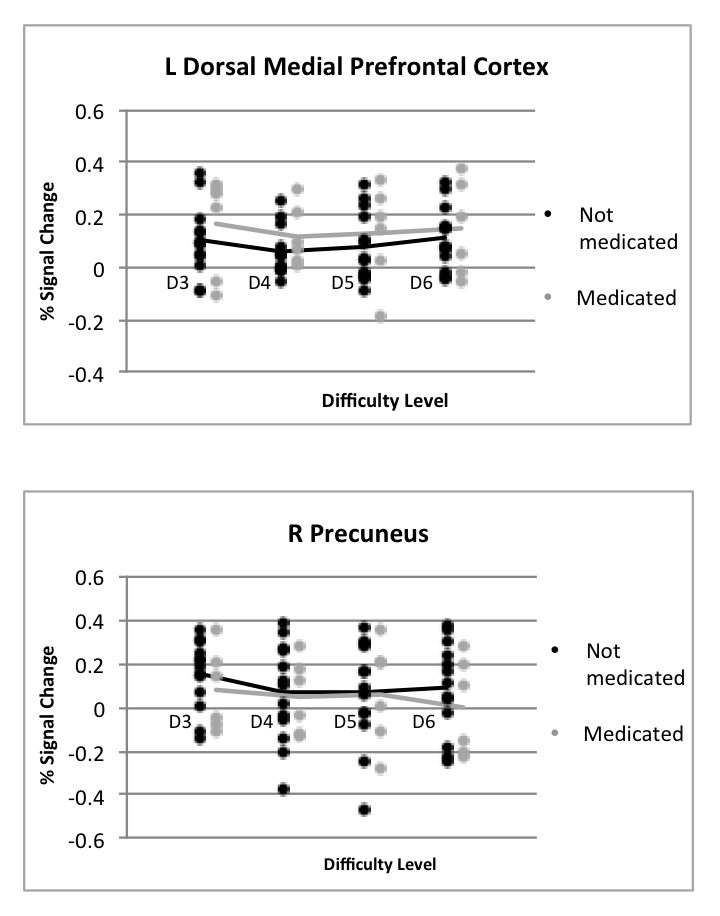

Supplement: Additional file 1 — fMRI data for medicated and non-medicated children with ASD. Percent signal change as a function of difficulty between task difficulty and baseline conditions in children with ASD who were on medication versus those who were not. Areas of the brain shown are from regions where children with ASD significantly differed from TD children in the linear trend analyses. Potential differences between medicated and non-medicated children with ASD were also examined statistically using the FSL FEAT, and no significant differences were found between children with ASD who were and were not on medication. However, due to low N (only six subjects on medication), this statistical test may not be reliable. Therefore scatter plots were created to visually examine the data for significant group differences, and this reaffirmed that medication does not appear to affect the findings. [file 1866-1955-6-19-S1.png]

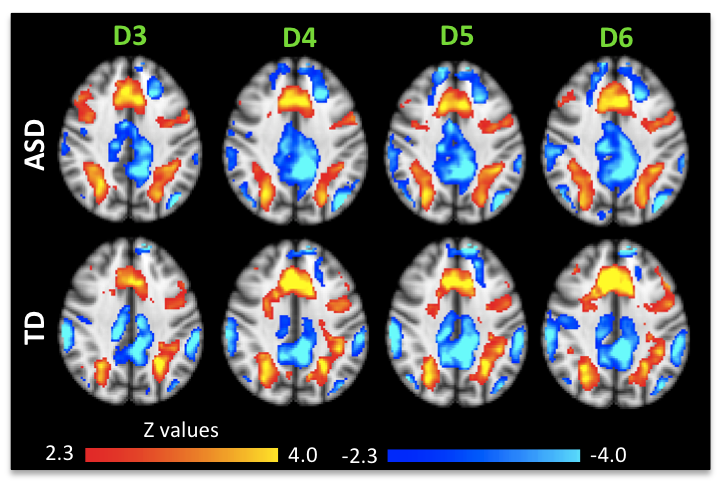

Supplement: Additional file 2 — Individual group activation maps for children with ASD and typically developing children at all levels (D3 to D6) of CMT. Significant activations using cluster-based thresholding determined by Z > |2.3| and a corrected cluster significance threshold of p = 0.05. Areas in red and blue depict regions with significantly higher and lower BOLD signal than baseline, respectively. Between-group comparisons showed no areas of significant difference between children with and without ASD at any single difficulty level. [file 1866-1955-6-19-S2.png]
